# Gastroesophageal reflux disease is a risk factor for sputum production in the general population: the Nagahama study

**DOI:** 10.1186/s12931-020-01601-y

**Published:** 2021-01-06

**Authors:** Chie Morimoto, Hisako Matsumoto, Tadao Nagasaki, Yoshihiro Kanemitsu, Yumi Ishiyama, Hironobu Sunadome, Tsuyoshi Oguma, Isao Ito, Kimihiko Murase, Takahisa Kawaguchi, Yasuharu Tabara, Akio Niimi, Shigeo Muro, Fumihiko Matsuda, Kazuo Chin, Toyohiro Hirai

**Affiliations:** 1 Department of Respiratory Medicine, Graduate School of Medicine, Kyoto University, 54, Kawahara-cho, Shogoin, Sakyo-ku, Kyoto, 606-8507 Japan; 2 Division of Respiratory Medicine, Department of Medical Oncology and Immunology, Nagoya City University School of Medical Sciences, Nagoya, Japan; 3 Department of Respiratory Care and Sleep Control Medicine, Graduate School of Medicine, Kyoto University, Kyoto, Japan; 4 Center for Genomic Medicine, Graduate School of Medicine, Kyoto University, Kyoto, Japan; 5 Department of Respiratory Medicine, Nara Medical University, Nara, Japan

**Keywords:** Sputum production, Gastroesophageal reflux disease, Epidemiological study

## Abstract

**Background:**

Chronic sputum production in the general population is historically associated with clinical indices including male sex and smoking history. However, its relationship with gastroesophageal reflux disease (GERD), which may prove an underlying factor in sputum production, is unclear. We aimed to clarify factors associated with sputum production in the general population in cross-sectional and longitudinal manners.

**Methods:**

In the Nagahama study, a community-based cohort study, 9804 subjects were recruited between 2008 and 2010 (baseline assessment), 8293 of whom were followed from 2013 to 2015 (follow-up assessment). This study contained a self-completed questionnaire which included medical history, assessment of sputum production, and a frequency scale for symptoms of GERD. A Frequency Scale for Symptoms of Gastroesophageal Reflux Disease score of ≥ 8 was defined as GERD. In addition to the frequency of sputum production at each assessment, frequency of persistent sputum production defined as sputum production at both assessments was examined.

**Results:**

Frequency of sputum production was 32.0% at baseline and 34.5% at follow-up. Multivariable analysis demonstrated that sputum production at baseline was significantly associated with GERD [odds ratio (OR), 1.92; 95% confidence interval (CI) 1.73–2.13] and post-nasal drip (PND) (OR, 2.40; 95% CI 2.15–2.68), independent of other known factors such as older age, male sex and smoking history. These associations between sputum production and GERD or PND were also observed at follow-up. In longitudinal analysis, 19.4% had persistent sputum production and 12.3% had transient sputum production, i.e., at baseline only. Multivariable analysis for risk of persistence of sputum production revealed that persistent sputum production was associated with GERD and PND, in addition to the known risk factors listed above. The proportion of subjects with GERD at both assessments was highest among subjects with persistent sputum production.

**Conclusions:**

Cross-sectional and longitudinal analysis demonstrated an association in the general population between sputum production and GERD, as well as PND, independent of known risk factors. The presence of GERD should be assessed in patients complaining of sputum production.

## Introduction

Chronic sputum production is a troublesome symptom associated with respiratory diseases and is also reported in the general population [1, 2], with a prevalence ranging from 1.2 to 11.9%. Chronic sputum production may be defined as sputum production lasting for at least 3 months per year for more than 1 year. This definition of chronic sputum production can be used to identify chronic bronchitis, which is strongly associated with smoking, air pollution, and occupational exposure to dust or fumes. However, in clinical practice, we often encounter patients who have not been exposed to smoking or dust, yet who complain of sputum production. In developed countries, including Japan, air pollution has improved and smoking prevalence has decreased [3], which may have changed the epidemiology and risk factors of sputum production over the last few decades.

According to previous epidemiological studies, male sex, smoking history, and low socioeconomic status [1, 2, 4] are the established risk factors for sputum production. Recent studies have shown that gastroesophageal reflux disease (GERD) is a common comorbidity associated with exacerbations of lower airway diseases, including chronic obstructive pulmonary disease (COPD) [5–7] and asthma [8–11]. GERD may therefore represent another risk factor. Although GERD is thought to be associated with a dry cough [12, 13], it was shown to be an independent risk factor for productive cough in a population-based study [11], and was independently associated with the degree of sputum production in COPD patients [6], suggesting it likely influences sputum production. However, the association between GERD and sputum production in the general population remains unknown. If any such association is confirmed, GERD should be suspected in subjects with sputum production. In the present study, we aimed to clarify the factors associated with sputum production in the general population, focusing on the effects of GERD in both cross-sectional and longitudinal manners.

## Methods

### Study design and population

This study was based on data obtained from participants of the Nagahama Cohort for Comprehensive Human Bioscience (the Nagahama study). Subjects were recruited between 2008 and 2010 (baseline assessment) among apparently healthy residents without physical impairment, aged 30–74, from Nagahama City, which is a large rural city in the Shiga prefecture in central Japan. Subjects were followed-up between 2013 and 2015 (follow-up assessment). In total, 9804 subjects were recruited, of whom 8293 participated in follow-up assessment [14]. This community-based cohort study contained a self-completed questionnaire which included medical history, assessment of sputum production, and Frequency Scale for Symptoms of GERD (FSSG). Blood tests and pulmonary function test were also performed but not included for this specific analysis of sputum production.

This study was approved by the Ethics Committee of Kyoto University Graduate School and Faculty of Medicine, the Ethical Review Board of the Nagahama Study, and the Nagahama Municipal Review Board of Personal Information Protection. Written informed consent was obtained from all participants.

### Questionnaire

At baseline and follow-up assessments, symptoms, past medical histories, and smoking habits were assessed using a series of structured questionnaires. To assess sputum production, subjects responded to the following question:

- Do you produce sputum during the day? Patients could select from *alway*s*, sometimes*, or *never*.

The presence of sputum production was concluded in patients who answered *always* or *sometimes*. Persistent and transient sputum production was defined, respectively, as sputum production at both assessments and production at baseline assessment only. GERD [15] was evaluated using a FSSG, consisting of questions related to reflux and dyspeptic symptoms (Additional file 1: Table S1) [16], which correlates with the endoscopic grade of esophagitis and esophageal peristaltic pressures during dry swallow [17]. In this study, patients were diagnosed with GERD when total scores were ≥ 8 [15]. Post-nasal drip (PND), prolonged cough, stress, and medical histories, including asthma, COPD, and sinusitis were all also evaluated using a self-completed questionnaire. Prolonged cough was defined as a cough lasting ≥ 3 weeks [14]. At baseline only, stress was assessed with the question:

- Have you felt stress in the last year? Patients could select either *considerable stress, a certain amount of stress,* or *little-to-no stress*.

Stress was considered present in subjects who answered *considerable* or *a certain amount of stress*.

### Statistical analysis

All statistical analyses were performed using JMP Pro 12 (SAS Institute Inc., Tokyo, Japan). Associations with sputum production were performed using the Chi-squared test and t-test, and multivariable analysis was performed using logistic regression. In all instances, p < 0.05 was considered statistically significant.

## Results

Participant characteristics were divided according to sputum production at baseline and follow-up assessments (Table 1). The frequency of sputum production was 32.0% at baseline and 34.5% at follow-up. Subjects who complained of sputum production at baseline had a higher frequency of known risk factors including older age, male sex, smoking history, COPD, asthma, and sinusitis than those who did not (Table 2). Those with sputum production also had a higher frequency of GERD (FSSG ≥ 8) than those who did not (32.7% vs. 18.0%; p < 0.0001). In multivariable analysis, GERD was significantly associated with sputum production [odds ratio (OR), 1.92; 95% confidence interval (CI) 1.73–2.13], which was independent of the known risk factors listed above. When reflux symptoms and dyspeptic symptoms (Additional file 1: Table S1) were analyzed separately, each symptom was significantly associated with sputum symptoms (data not shown). PND was also more frequently observed in patients with sputum production than in those without, and was proven to be another independent risk factor (OR, 2.40; 95% CI 2.15–2.68) in multivariable analysis. These associations between sputum production and GERD or PND at baseline were also observed at follow-up (Additional file 1: Table S2).

**Table 1:** Participant demographics (baseline assessment)

Characteristics	All subjects n = 9804	Transient sputum production^a^ n = 1022	Persistent sputum production^a^ n = 1609	Sputum production at follow-up only^a,b^ n = 1256	No sputum production at both assessments^a^ n = 4406	p value among the four groups
Age, years	53.6 ± 13.4	53.6 ± 13.8	54.9 ± 13.3	53.7 ± 12.4	53.8 ± 12.9	0.01
Sex (male/female), %	32.8/67.2	40.6/59.4	50.4/49.6	30.8/69.2	23.7/76.3	< 0.0001
Sputum production (baseline/follow-up), %	32.0/34.5	100/0	100/100	0/100	0/0	–
Sputum production on awakening (baseline/follow-up), %	16.7/19.6	32.9/8.5	49.0/58.8	8.9/38.4	3.9/2.5	< 0.0001/< 0.0001
Smoking history (ex or current), %	35.0	40.9	49.8	33.4	26.1	< 0.0001
COPD, %	1.1	1.5	2.1	1.1	0.5	< 0.0001
Asthma, %	4.1	5.5	6.6	4.0	2.9	< 0.0001
Prolonged cough, %	10.4	14.7	18.3	10.4	7.0	< 0.0001
Sinusitis, %	10.2	12.5	15.7	10.5	7.9	< 0.0001
Post-nasal drip (baseline/follow-up), %	21.1/24.9	28.8/24.7	35.5/39.7	22.1/36.6	14.4/16.2	< 0.0001/< 0.0001
FSSG score ≥ 8 (baseline/follow-up), %	22.7/22.0	26.7/23.4	36.5/36.3	25.2/28.1	15.8/14.8	< 0.0001/< 0.0001

Data is presented as mean ± SD. Data at baseline assessment is presented unless otherwise specified.  Transient; sputum production at baseline only. Persistent; sputum production at both baseline and follow-up assessments

*COPD* chronic obstructive pulmonary disease, *FSSG* Frequency Scale for Symptoms of Gastroesophageal Reflux Disease

^a^ among 8293 participants who were followed from 2013 to 2015

^b^ among 8292 for post-nasal drip and FSSG score

**Table 2:** Factors associated with sputum production at baseline: comparative and multivariable analyses

Baseline factors	Sputum + n = 3139	Sputum − n = 6665	p value	OR (95% CI) for sputum production	p value
Age, year^a^	54.0 ± 13.9	53.4 ± 13.2	0.04	1.10 (1.06–1.14)	< 0.0001
Male sex, %	47.0	26.1	< 0.0001	2.34 (2.07–2.65)	< 0.0001
BMI, kg/m^2^	22.5 ± 3.4	22.2 ± 3.2	< 0.0001	1.00 (0.98–1.01)	0.87
Smoking history (ex or current), %	47.6	29.0	< 0.0001	1.49 (1.33–1.68)	< 0.0001
COPD, %	2.2	0.6	< 0.0001	2.21 (1.44–3.38)	0.0003
Asthma, %	6.3	3.1	< 0.0001	1.53 (1.22–1.90)	0.0002
Prolonged cough, %	16.6	7.6	< 0.0001	2.31 (2.00–2.66)	< 0.0001
Allergic rhinitis, %	37.7	34.0	0.0003	1.02 (0.92–1.12)	0.76
Sinusitis, %	14.1	8.3	< 0.0001	1.39 (1.20–1.60)	< 0.0001
Post-nasal drip, %	32.3	15.9	< 0.0001	2.40 (2.15–2.68)	< 0.0001
Diabetes mellitus, %	7.0	4.4	< 0.0001	1.25 (1.02–1.52)	0.03
FSSG score ≥ 8, %	32.7	18.0	< 0.0001	1.92 (1.73–2.13)	< 0.0001
Stress, %	74.3	71.1	0.0007	1.30 (1.17–1.45)	< 0.0001

*OR* odds ratio, *CI* confidence interval, *BMI* body mass index, *COPD* chronic obstructive pulmonary disease, *FSSG* Frequency Scale for Symptoms of Gastroesophageal Reflux Disease

^a^Age per 10-year increase

For multivariable analysis

Among 8293 participants assessed at both baseline and follow-up, 1609 participants (19.4%) had persistent sputum production, 1022 (12.3%) had transient production (i.e., at baseline only), 1256 (15.1%) had sputum production at follow-up only, and 4406 (53.1%) had production at neither assessment (Table 1). GERD and PND at baseline were more frequently observed in participants with persistent sputum production than in those with transient production (Table 3). Multivariable analysis for risk of persistence of sputum production, performed in the 2631 participants with sputum production at baseline, revealed that persistent sputum production was significantly associated with GERD and PND at baseline, in addition to older age, male sex, and smoking history (Table 3). Even when analysis was confined only to subjects free from COPD and asthma, similar associations were observed between persistent sputum production and GERD or PND (data not shown). The association between persistent sputum production and GERD at baseline was also confirmed by the subgroup analysis of subjects without PND (Table 4).

**Table 3:** Factors associated with persistent sputum production at baseline: comparative and multivariable analyses

Baseline factors	Persistent sputum n = 1609	Transient sputum n = 1022	p value	OR (95% CI) for persistent sputum	p value
Age, year^a^	54.9 ± 13.3	53.6 ± 13.8	0.01	1.10 (1.04–1.17)	0.002
Male sex, %	50.4	40.6	< 0.0001	1.35 (1.10–1.65)	0.004
BMI, kg/m^2^	22.6 ± 3.3	22.5 ± 3.4	0.4		
Smoking history (ex or current), %	49.8	40.9	< 0.0001	1.28 (1.04–1.56)	0.02
COPD, %	2.1	1.5	0.3		
Asthma, %	6..6	5.5	0.2		
Allergic rhinitis, %	39.0	37.0	0.3		
Sinusitis, %	15.7	12.5	0.02	1.17 (0.92–1.48)	0.2
Post-nasal drip, %	35.5	28.8	0.0003	1.40 (1.16–1.67)	0.0003
Diabetes mellitus, %	7.1	6.5	0.5		
FSSG scores ≥ 8, %	36.5	26.7	< 0.0001	1.58 (1.32–1.88)	< 0.0001
Stress, %	74.5	73.3	0.5		

Transient; sputum production at baseline only. Persistent; sputum production at both baseline and follow-up assessments

*OR* odds ratio, *CI* confidence interval, *BMI* body mass index, *COPD* chronic obstructive pulmonary disease, *FSSG* Frequency Scale for Symptoms of Gastroesophageal Reflux Disease

^a^Age per 10-year increase

For multivariable analysis

**Table 4:** Subgroup analysis for persistent sputum production in subjects without post-nasal drip: multivariable analysis (N = 1766)

Baseline factors	Persistent sputum production		
	OR	95% CI	p value
Age, per 10-year increase	1.12	1.04–1.21	0.002
Male sex	1.37	1.07–1.74	0.01
Smoking history (ex or current)	1.32	1.04–1.68	0.03
FSSG scores ≥ 8	1.68	1.34–2.11	< 0.0001

*FSSG* Frequency Scale for Symptoms of Gastroesophageal Reflux Disease

**Fig. 1:**
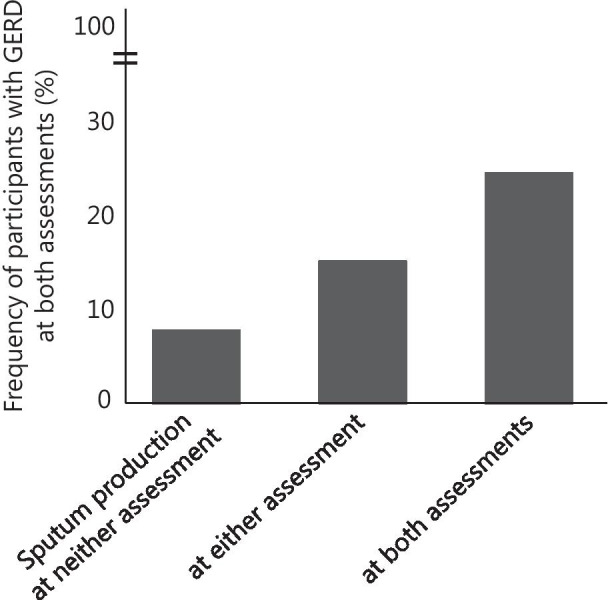
Association between persistent sputum production and GERD in longitudinal assessment. Frequency of subjects with GERD (FSSG ≥ 8) at both assessments, in subjects with sputum production at neither assessment (N = 4406), at either assessment (N = 2278), and at both assessments (N = 1609). p < 0.0001 (Chi-squared test)

Finally, persistent symptoms due to GERD and PND were assessed to confirm their concordance with sputum production. Frequencies of participants with GERD at both assessments, baseline only, and neither were 13.1%, 9.5% and 68.4%, respectively. The proportion of subjects with GERD at both assessments was highest among subjects with persistent sputum production (p < 0.0001) (Fig. 1). Frequencies of participants with PND at both assessments, baseline only, and neither were 12.7%, 8.7% and 66.4%, respectively. Similarly, the proportion of subjects with PND at both assessments was highest among subjects with persistent sputum production (p < 0.0001, data not shown).

## Discussion

To the best of our knowledge, this is the first study that demonstrated longitudinally that GERD was associated with sputum production. In both cross-sectional and longitudinal analysis, this association was independent of known risk factors including older age, male sex, and smoking history.

In the present study, frequencies of sputum production were 32.0% at baseline and 34.5% at follow-up, both of which are greater than the reported prevalence of chronic sputum production (1.2–11.9%) [2, 4, 18]. This difference may be due to differences in how sputum production is defined. In each assessment, sputum production was defined regardless of duration, which may have included transient production. Nevertheless, sputum production with an unrestricted duration is still worth investigating, considering commonly used COPD questionnaires like the COPD assessment test (CAT) and cough and sputum asking questionnaire (CASA-Q) assess current sputum production and sputum production within the past 7 days, respectively. Previous studies using CAT and CASA-Q in patients with COPD identified both GERD and PND as risk factors for sputum production [6], consistent with the cross-sectional findings of the present study.

Previously, a population-based study revealed that subjects with severe and recurrent reflux symptoms were more likely to experience daily productive cough than those without reflux symptoms (OR, 1.9; 95% CI 1.7–2.2) [11]. Consistently, this study showed that OR of GERD for sputum production at baseline was 1.92 (95% CI 1.73–2.13). Furthermore, when persistence of sputum production was assessed in a longitudinal manner, frequency of persistent sputum production was 19.4%, with GERD at baseline contributing significantly and independently from known risk factors of older age, male sex and smoking history [2, 4, 18]. There was also a corresponding increase in the frequency of persistent sputum production and persistent symptoms due to GERD. These findings from cross-sectional and longitudinal analysis strongly suggest an association between sputum production and GERD. Regarding underlying mechanisms, several experimental studies have suggested that the activation of parasympathetic signaling by distal esophageal acid induces airway mucus hypersecretion [19–21]. In a GERD mouse model, intra-esophageal HCl instillation resulted in microvascular leakage and marked inflammatory cell infiltration into the airways and peribronchial areas [20], which was inhibited by use of muscarinic receptor antagonists and bilateral vagotomy [20, 21]. In addition to acid exposure, gastrointestinal dysmotility is an important factor in GERD, one which may contribute to sputum production.

Another important risk factor for sputum production in this study was PND. Among several different risk factors, the OR of PND for sputum production in cross-sectional and longitudinal analyses was highest or second highest. Although difficult to prove, subclinical sinusitis or sinobronchial syndrome accompanying PND may have affected sputum production in the current study. This speculation is supported by a COPD study which demonstrated that sputum production was associated not only with PND but also with other nasal symptoms [22]. One concern is that subjects in the present study might have failed to distinguish sputum production from PND and may have expressed excess mucus in their throat and mouth as sputum production regardless of origin. This is one limitation to the study; nonetheless, it also emphasizes the need for careful interviewing in clinical practice to assess sputum production and distinguish it from PND.

Another limitation of the present study is that most of the data, including frequency of sputum production, was based on self-completed questionnaires. Therefore, the group of subjects with sputum production might have included those with sputum sensation at the throat only. However, consistent findings regarding prevalence of sputum and contributing factors at baseline and follow-up may compensate for this limitation. Next, we focused on sputum production during the day in this study. However, we did also analyze sputum production on awakening (Table 1), confirming similar associations between morning sputum production and GERD or PND (data not shown). Finally, endoscopic examinations by specialists were not conducted in this study to assess the presence of GERD. However, the prevalence of GERD in this study was 23%, similar to previously reported in Japan [23, 24]. Additionally, the reflux score, which consists of FSSG together with the dyspepsia score, is more useful in distinguishing GERD from gastric and duodenal ulcer, and functional dyspepsia than total score of FSSG [25]. Indeed, the reflux score was significantly associated with sputum production in this study. Furthermore, non-erosive reflux disease, which shows no visible esophageal mucosal injury, is the most common phenotypic presentation of GERD [26]; thus, endoscopic examination is not necessarily required to define GERD. In addition, the FSSG used in this study is a validated measure of GERD; other epidemiological studies on GERD prevalence are also based on GERD-related questionnaires [8, 27].

## Conclusions

The present study demonstrates an association in the general population between sputum production and GERD, as well as PND, independent of known risk factors. The presence of GERD should therefore be assessed in patients complaining of sputum production.

## Supplementary Information

**Additional file 1:** **Table S1.** Frequency Scale for the Symptoms of Gastroesophageal Reflux Disease (FSSG) questionnaire (adapted from references [15, 16]). **Table S2.** Factors associated with sputum production at follow-up assessment: multivariable analysis.

## Data Availability

The data generated and/or analyzed during the current study are not publicly available due to privacy policies but are available from the corresponding author upon reasonable request.

## Appendix

The Nagahama study group executive committee comprises the following individuals: Yasuharu Tabara, Takahisa Kawaguchi, Kazuya Setoh, Yoshimitsu Takahashi, Shinji Kosugi, Takeo Nakayama, and Fumihiko Matsuda from the Center for Genomic Medicine, Kyoto University Graduate School of Medicine (YaT, TK, KS, FM); Department of Health Informatics (YoT, TN); Department of Medical Ethics and Medical Genetics (SK), Kyoto University School of Public Health.

## Abbreviations

CASA-Q: Cough and sputum asking questionnaire
CAT: COPD assessment test
CI: Confidence interval
COPD: Chronic obstructive pulmonary disease
FSSG: Frequency scale for symptoms of GERD
GERD: Gastroesophageal reflux disease
OR: Odds ratio
PND: Post-nasal drip
