# Supplementary material for: Gastroesophageal reflux disease is a risk factor for sputum production in the general population: the Nagahama study
Source: Respir Res. 2021 Jan 6;22:6. doi: 10.1186/s12931-020-01601-y (PMC7788913; doi:10.1186/s12931-020-01601-y)
Supplement: Supplementary file 1 — Additional file 1: Table S1. Frequency Scale for the Symptoms of Gastroesophageal Reflux Disease (FSSG) questionnaire (adapted from references [15, 16]). Table S2. Factors associated with sputum production at follow-up assessment: multivariable analysis. [file 12931_2020_1601_MOESM1_ESM.docx]

Table S1. Frequency Scale for the Symptoms of Gastroesophageal Reflux Disease (FSSG) questionnaire (adapted from reference 15 and 16).

| Question | | Frequency | | | | |
| --- | --- | --- | --- | --- | --- | --- |
|  |  | Never | Occasionally | Sometimes | Often | Always |
| 1 | Do you get heartburn? | 0 | 1 | 2 | 3 | 4 |
| 2 | Does your stomach get bloated? | 0 | 1 | 2 | 3 | 4 |
| 3 | Does your stomach ever feel heavy after meals? | 0 | 1 | 2 | 3 | 4 |
| 4 | Do you sometimes subconsciously rub your chest with your hand? | 0 | 1 | 2 | 3 | 4 |
| 5 | Do you ever feel sick after meals? | 0 | 1 | 2 | 3 | 4 |
| 6 | Do you get heartburn after meals? | 0 | 1 | 2 | 3 | 4 |
| 7 | Do you have an unusual (e.g., burning) sensation in your throat? | 0 | 1 | 2 | 3 | 4 |
| 8 | Do you feel full while eating meals? | 0 | 1 | 2 | 3 | 4 |
| 9 | Do some things get stuck when you swallow? | 0 | 1 | 2 | 3 | 4 |
| 10 | Do you get bitter liquid (acid) coming up into your throat? | 0 | 1 | 2 | 3 | 4 |
| 11 | Do you burp a lot? | 0 | 1 | 2 | 3 | 4 |
| 12 | Do you get heartburn if you bend over? | 0 | 1 | 2 | 3 | 4 |

Questions related to reflux score (#1, 4, 6, 7, 9, 10, and 12)

Questions related to dyspepsia score (#2, 3, 5, 8, and 11)

Table S2

Factors associated with sputum production at follow-up assessment: multivariable analysis

|  | Sputum production at the follow-up | | |
| --- | --- | --- | --- |
| Factors at the follow-up | OR | 95% CI | p value |
| Age, per 10-year increase | 1.10 | 1.05–1.14 | <0.0001 |
| Male sex | 1.80 | 1.58–2.05 | <0.0001 |
| BMI, kg/m^2^ | 1.01 | 0.99–1.02 | 0.41 |
| Smoking history (ex or current) | 1.46 | 1.28–1.66 | <0.0001 |
| COPD | 1.42 | 0.92–2.17 | 0.11 |
| Asthma | 1.84 | 1.47–2.30 | <0.0001 |
| Prolonged cough | 2.02 | 1.76–2.33 | <0.0001 |
| Allergic rhinitis | 1.13 | 1.02–1.26 | 0.02 |
| Sinusitis | 1.33 | 1.14–1.56 | 0.0003 |
| Post-nasal drip | 2.50 | 2.23–2.81 | <0.0001 |
| Diabetes mellitus | 1.01 | 0.84–1.22 | 0.92 |
| FSSG score ≥8 | 2.12 | 1.89–2.38 | <0.0001 |

OR, odds ratio; CI, confidence interval; BMI, body mass index; COPD, chronic obstructive pulmonary disease; FSSG, Frequency Scale for Symptoms of Gastroesophageal Reflux Disease.
